# Wild mushroom- an underutilized healthy food resource and income generator: experience from Tanzania rural areas

**DOI:** 10.1186/1746-4269-9-49

**Published:** 2013-07-10

**Authors:** Donatha D Tibuhwa

**Affiliations:** 1 Department of Molecular Biology and Biotechnology (MBB), University of Dar es Salaam, PO Box 35179, Dar es Salaam, Tanzania

**Keywords:** Mycological knowledge, Wild edible mushrooms, Rural economy, Rural areas

## Abstract

**Background:**

This study documents the use of a wild edible mushroom (WEM) in Tanzania rural areas and assesses its significance as a source of healthy food and income for the disadvantaged rural dwellers.

**Methodology:**

The data was gathered through local market surveys in order to conventionally identify different common WEM taxa using a semi-structured interview and it involved 160 people comprised of WEM hunters, traders and consumers. The collected data covered the information on where, how, when and who was the principal transmitter of the mycological knowledge learned and the general information on their market and values.

**Results:**

Results show that mushroom gathering is gender oriented, dominated by women (76.25%) whereas men account for 23.75%. Women possess vast knowledge of mushroom folk taxonomy, biology and ecology and are therefore the principal knowledge transmitters. It was also found that learning about WEM began at an early age and is family tradition based. The knowledge is acquired and imparted by practices and is mostly transmitted vertically through family dissemination. The results also revealed that 75 WEM species belong to 14 families sold in fresh or dry form. The common sold species belonged to the family Cantharellaceae (19) followed by Rusullaceae (16) and Lyophyllaceae (13), respectively. Collectors residing near miombo woodland may harvest 20–30 buckets (capacity 20 liters) and the business may earn a person about $400–900 annually.

**Conclusion:**

This finding envisages the purposeful strengthening of WEM exploitation, which would contribute significantly in boosting the rural income/economy and reduce conflicts between community and forest conservers. The activity would also provide alternative employment, improve food security to rural disadvantaged groups especially women and old people hence improve their livelihood.

## Introduction

Local mycological knowledge which includes the use of mushroom as food, medicinal application, recreational objects, beliefs and myths, as well as income generating activity to poor households is well documented in different parts of the world [1-6]. The information on how to recognize and differentiate between edible and none edible mushroom depends largely on folk taxonomy. Folk taxonomy is the classification of organisms on the basis of cultural tradition which uses vernacular naming system [5]. The folk taxonomic knowledge provides the tool for communication and information survival from one generation to another. Recently, there have been increasing interests in mushrooms utilization worldwide. They are taken as either taste food or because of their special biochemical compositions, with significant contents of antioxidant compounds, proteins, carbohydrates, lipids, enzymes, minerals, vitamins and water. These essential components attract more attention as functional health promoters and in development of drugs and nutraceuticals [7-11].

Although there is increasing interest in picking wild mushrooms in both developed and developing countries for use as food and medicinal applications, there is scanty documentation of social economic and environmental implications. The statistical information on the amount of collected WEM and its associated values is patchy and often unreliable [12]. The only area with good statistical information of WEM commercial picking is the Pacific North-Western United States [2,13]. For example, in Europe few studies of commercial wild mushroom gathered includes that of Dyke and Newton [14] who surveyed the pickers, buyers and landowners in order to assess their sustainability in Scotland. In Northern Spain, De Roman and Boa [15] analyzed how gathering and marketing of *Lactarius deliciosus* affected a small rural community whereas Cai *et al*. [16] recently documented the emergence of commercial wild mushroom harvesting in Eastern Finland.

Like many other developing countries, the gross national income (GNI) per capita of Tanzania was estimated at $340 by World Bank [17]. Most of the poorest group (87%) lives in rural areas with their economic activities based on subsistence agriculture, which depends heavily on one crop such as maize, cassava, beans, millet or sorghum. The changing climate has led to excessive drought which threatens the traditional crops and has consequently contributed to famine and hunger. In order to promote rural development, there is a need to diversify rural income sources and increase job opportunities.

Wild edible mushrooms are among the None Wood Forest Products not well documented in many countries including Tanzania. In fact mushroom forming fungi are poorly collected, sparingly studied and relatively underutilized in the country. For example, there is no efficient information on how much is harvested, no market orders and channels as well as general awareness regarding the income generation potential and its contribution to food security. With an exception of the comprehensive work done by Härkönen et al. [18,19] which documented more than 100 species of mushroom in Tanzania and Tibuhwa [5] who documented the Folk taxonomy and use of mushrooms in communities around Ngorongoro and Serengeti National Park, Tanzania; mushroom researches in the country have been generally conducted on the mushroom biology sciences, taxonomy, diversity and the bioactive compounds from mushrooms [19-24]. There is no purposeful effort by the Ministry of Agriculture in Tanzania to update the status of mushroom as a crop. This leads to a lack of documentation and reports on the amount of mushroom produced, harvested, exported and imported which would have helped in establishing the market value chain of mushroom dynamics in the country.

In this study, ethno-mycology and wild edible mushroom resources exploitation for healthy food income and generation, experience from marginal rural areas in Tanzania is presented.

## Materials and methods

### Study site

**Figure 1:**
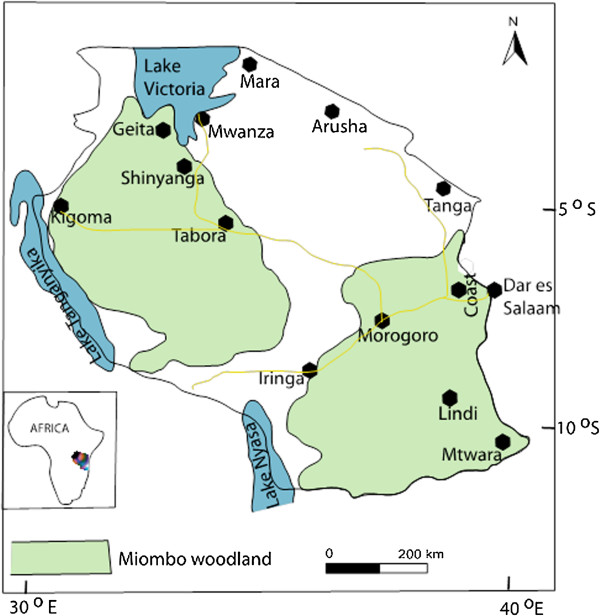
Map of Tanzania showing the studied sites.

The study was conducted in six zones (Figure 1) viz: Western part (Kigoma and Tabora), Lake zone (Geita, Mwanza and Shinyanga), North eastern (Mara and, Arusha -Ngorongoro), Southern coast (Lindi and Mtwara) and Northern coast (Coastal, Tanga, Dar es Salaam). The study areas composed of mixed forest including the miombo woodland dominating in the Southern highlands, Southern coast and Lake Zone. The North coast part was mainly dominated by indigenous natural tropical forest while the North eastern regions were characterized by thorn woodland trees species in the genera *Acacia* Mill., *Commiphora* Jacq., *Ficus* L., *Combretum* Loefl. and *Podocarpus* Persoon and extensive grass plains [5].

### Data acquisition

A local field survey was carried in the six zones which are administratively divided into twelve different regions in the county (Figure 1). Twenty six ethnic groups were involved in the survey and are engaged in subsistence agriculture, livestock keeping, artisanal fishing, hunting, gathering and small mining (Table 1). A semi-structured interview was used where by a list of questions intending to gather similar information from different interviewee were asked face-to-face. The interview involved 160 people who were from three main groups as detailed below. The first groups comprised of wholesalers who were local mushroom pickers. They are the ones who go to the forest/fields to pick the mushroom and sell them to retail traders. The second group was the business people who sell mushroom in local markets while the third group comprised of consumers who eat mushrooms by either direct collecting them from nearby forest and fields, or buy them from local markets and street mushroom vendors. The interviews were conducted from year 2004 to late 2011 as a result of long time engagement of the author with mushroom research.

**Table 1:** Studied ethnic groups settlement and their main economic activities

**Country zone**	**Settlement region**	**Dominant tribes**	**Economic activities**
**Western part**	Kigoma	Ha	agriculture, livestock keeping, fishing and gathering
	Tabora	Nyamwezi, Manyema	agriculture, livestock keeping and gathering
**Lake zone**	Geita	Sukuma, Zinza, Subi	agriculture, livestock keeping, fishing, gathering, mining
	Mwanza	Sukuma, Kerewe, Zinza	fishing, agriculture and gathering
	Shinyanga	Sukuma, Nyamwezi, Nyantuzu	livestock keeping, fishing, mining agriculture and gathering
**North eastern**	Mara-Serengeti	Kurya, Jita	livestock keeping, fishing, hunting and gathering
	Arusha-Ngorongoro	Maasai, Sonjo	livestock keeping, hunting and subsistence agriculture
**Southern coast part**	Lindi	Ngindo	agriculture, fishing and gathering
	Mtwara	Makonde, Mwela, Makua, Yao	agriculture and gathering
**Southern highlands**	Iringa	Hehe, Bena, Kinga	agriculture, livestock keeping, hunting and gathering
**Northern coast part**	Coast	Zaramo, Ngindo, Kwele	fishing, subsistence agriculture and gathering
	Tanga	Zigua, Bondei	agriculture and gathering
	Dar es Salaam	Zaramo, Ngindo	fishing, subsistence agriculture and gathering

Whole sellers who are mainly pickers were questioned about their general knowledge on mushroom gathering activities, any ethno-mycological information they know, any concerns pertaining to the picking of wild mushrooms as well as related socio-economic characteristics.

Face to face to interviews were preferred to because the preliminary method of questionnaires proved failure as many candidates were not ready to spend their time in filling the form. Some of the interviewees were old people who did not know how to write and read but agreed to respond to oral questions. The demographic information was part of the interview questions as summarized in Table 2 as well as ethno-mycological information including folk taxonomy, market contemplation such as the type of mushrooms they sell? Where do they get them, how much do they collect and earn per day/ per season? How many people sell mushrooms? What time do they spend in gathering mushrooms? Who buys their mushrooms? If they don’t sell all they collect in a day, how do they preserve them and for how long?

**Table 2:** Demographic features of the informants (n= 160)

	**Frequency**	**Percentage**		**Frequency**	**Percentage**
**Gender**			**Level of education**		
Male	38	23.75%	No formal education	28	17.50%
Female	122	76.25%	Primary education	101	63.13%
**Marital status**			Adult education	8	5.00%
Married	107	66.87%	Secondary education	18	11.25%
Single	32	20.00%	College education	4	2.50%
Widow	21	13.13%			
**Age**			Employment status		
Between 12-17	22	13.75%	Employed	2	1.25%
Between 18-35	35	21.88%	Farmer/pastoralist	151	94.37%
Between 36-50	46	28.75%	Retired	7	4.38%
More than 50	57	35.63%			

### Data analysis

Specific demographic features and cross-relationships of the participants related to their general participation in WEM business, knowledge on the actual use and folk taxonomy were evaluated descriptively (frequency and percentages) using a Statistical Package for Social Sciences [25] Program Version 15.0.

## Results and discussion

### Wild edible mushroom collection and folk taxonomy

**Figure 2:**
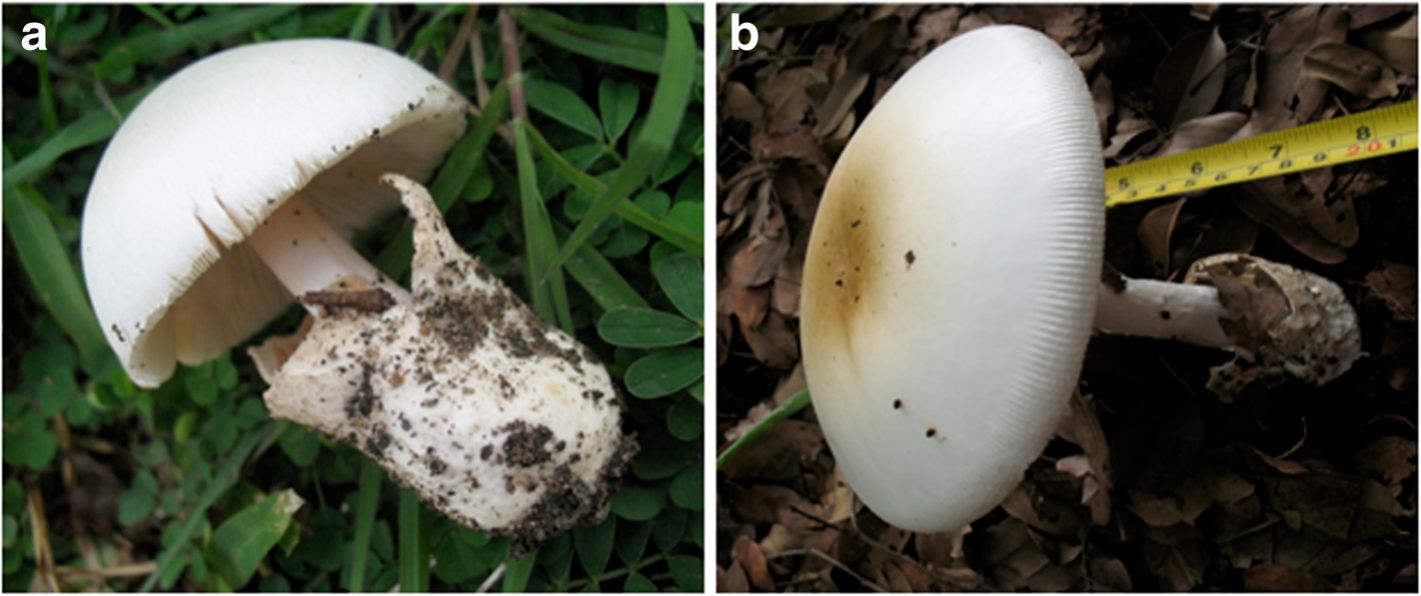
**Wild mushroom species which superficially look similar: (a) A delicious edible *Volvariella volvacea *(paddy straw mushroom); (b) A deadly poisonous *Amanita phalloides *(death cap).** (All photo taken by Tibuhwa DD in the field).

The study findings show that in Tanzania, public markets mushroom vendors who are typically indigenous women and children, eat/ sells 75 species of WEM (Table 3). The species belong to 14 families with the commonly sold species belonging to the family Cantharellaceae (19) followed by Rusullaceae (16) and Lyophyllaceae (13) respectively. The least sold species were from families represented by Pluteaceae, Suillaceae, Tremellaceae and Schizophyllaceae. The result also shows that unlike plant gathering, where the location of organisms is more predictable, mushroom are ephemeral in nature, fruit out only during the rain season. Nevertheless, not all mushrooms are edible; some are deadly poisonous and superficially look very similar to edible species (Figure 2). Because of seasonal fruiting, superficial similarity of poisonous and edible species, wild mushroom collectors must have folk taxonomy knowledge especially on how to characterize and identify mushroom of their interest.

**Table 3:** Common wild edible mushroom species consumed and sold in local markets

**S/N**	**Group based on DNA Bar Code of Life**	**Family**	**Scientific names**	**Commonly used folk names**
1	Euagarics Clade	Lyophyllaceae	*Termitomyces microcarpus* (Berk. and Broome) R. Heim	Kurya-Bitighose, Bena-unyonso, Hehe-Unyakigulu, Ngindo-Korowele; Nyamwezi-Kansolele & Busolele,Sukuma-Bumegere & Butuya
2	"	"	*Termitomyces titanicus* Pegler and Piearce	Kurya-Lyugu, Ha-Bhoba, Masaai-Ormambuli
3	"	"	*Termitomyces aurantiacus* (R. Heim) R. Heim	Ha-bhoba, Kurya-vihungumururyo, Sambaa-Vingongo,Bena-Widungu, Bondei-Kingong’ongo, Hehe-Videungwe
4	"	"	*Termitomyces clypeatus* R. Heim	Swahili-Kayeye hudhurungi, Ngindo-Lukuu, Kurya-Vihungumururyo
5	"	"	*Termitomyces eurhizus* (Berk.) R. Heim	Kurya-lyugu, Masaai-Ormambuli, Hehe-Chova, Pare-Kichoga cha ngombe, Kinga- Usumba
6	"	"	*Termitomyces le-testui* (Pat.) R. Heim	Kurya-lyugu, Masaai-Ormambuli, Swahili-mkufu, Nyambo-Nyamukundi, kwere-Nembo, Bondei & Sambaa-Kitundwi, Zaramo-Ng’uvu and Tembo
7	"	"	*Termitomyces mammiformis* R. Heim	
8	"	"	*Termitomyces umkowaan* (Cooke and Massee) D.A. Reid	Kurya-Amugu
9	"	"	*Termitomyces tylerianus* Otieno	Kurya-Vihungumururyo, Ngindo-Lukuu Swahili-Kiyoga mchwa laini
10	"	"	*Termitomyces saggitiformis* (Kalchbr. and Cooke) D.A. Reid	Kurya-Vihungumururyo
11	"	"	*Termitomyces singidensis* Saarim.and Härk.	Swahili-Impora, Hehe-Witali
12	"	"	*Termitomyces robustus* (Beli) Heim	Ngindo-Lukuu mkubwa
13	"	"	*Termitomyces striatus* (Beeli) R. Heim	Hehe-Vidungwe, Sambaa-Vigong’ongo, Bena-Widungu
14	"	Agaricaceae	*Agaricus campestris* L.:Fr.	Kurya-Nyankobhiti, Sambaa-fufu
15	"	"	*Agaricus bisporus* (J.E. Lange) Imbach	Kurya-Nyankobhiti, Sambaa-fufu
16	"	"	*Coprinus disseminatus* (Pers.) Gray	Swahili-Uyoga mkonge
17	"	"	*Coprinus comatus* (O.F. Müll.) Pers	Swahili-Uyoga mkonge
18	"	Pleurotaceae	*Pleurotus sajor-caju* (Fr.)	Swahili-Mangaha, Sambaa-Mangaha, Manga & Maangaa
19	"	"	*Pleurotus eryngii* (DC.) Quél.	Swahili-Mamama
20	"	"	*Pleurotus djamor* (Rumph. ex Fr.)	Swahili- Mamama, Sambaa-Mamama & Mameno
21	"	"	*Pleurotus tuber-regium* (Rumph. ex Fr.) Singer	Swahili-Mamama tunguu
22	"	"	*Macrolepiota procera* (Scop.) Singer	Ha-bhoba, Kurya-Nyankobhiti
23	"	Pluteaceae	*Volvariella volvacea* (Bull.) Singer	Swahili-Uyogambuyu kifuko, Yao-Ubuyu
24	"	Physalcriaceae	*Armillaria mellea* (Vahl) P. Kummer	Sambaa-Manjurugu
25	"	"	*Armillaria heimii* Pegler	Sambaa-Manjurugu
26	"	Amanitaceae	*Amanita loosi* Beeli	Hehe-Ulelema & Wilelema, Bena-Wilelemi
27	"	"	*Amanita mafingaensis* Härk. and Saarim.	Hehe-Wigwingwi, Swahili-Uwingwingwikahawia
28	"	"	*Amanita tanzanica* Härk. and Saarim*.*	Bena-Ugongoli & wigongoli, Yao-nakajongoo, Hehe-Wigwingwi
29	"	"	*Amanita masasiensis* Härk. and Saarim*.*	Bena- Ugongoli & Wigongoli, Hehe-wigwingwi,
30	Russuloid Clade	Rusullaceae	*Rusulla roseoviolacea* Quél.	Bena-Unyambete, Mmmeng’enyevu magamba
31	"	"	*Rusulla roseovelata* Buyck	Swahili-Mmeng’enyu zambarau, Hehe-unyamikwe
32	"	"	*Rusulla hiemisilvae* Buyck	Swahili-Mtundu ukanda, Hehe-Unyamikwe
33	"	"	*Rusulla congoana* Pat.	Hehe-unyamikwe, Nyamwezi-mnyitundu
34	"	"	*Rusulla ciliata* Buyck	Nyamwezi- utyelele, Sumbwa-Buntelele
35	"	"	*Rusulla compressa* Buyck	Bena-Widungu, Nyamwezi-Busegese Swahili-Damu ya mzee, Sukuma-Butundutundu
36	"	"	*Rusulla cellulata* Buyck	
37	"	"	*Lactarius densifolius* Verbeken and Karhula	Hehe-Unyakuwemba, Bena-wifimi Swahili-Uyoga maziwa tamu
38	"	"	*Lactarius heimii* Verbeken	Sukuma-Kansalage, Nyamwezi-Wikese, Hehe- Unyakuvemba
39	"	"	*Lactarius kabansus* Pegler	Nyamwezi-Umpalala, Hehe-wisiga
40	"	"	*Lactarius luteolus* Peck Bull.	Hehe-Unyakuwemba, Bena- Unyamalagata
41	"	"	*Lactarius medusae* Verbeken	Hehe-Unyakuwemba
42	"	"	*Lactarius pumilus* Verbeken	Swahili-Uyoga maziwa kibete, Hehe-Unyakuwemba
43	"	"	*Lactarius tanzanicus* Karhula and Verbeken	Ngindo-Uyoga ulambo, Yao-Uyoga mchenga
44	"	"	*Lactarius volemoides* (Fr.)	Bena-Wunyamagulu
45	"	"	*Lactarius xerampelinus* Karhula and Verbeken	Sukuma-Bushikoba, Hehe-wimenda, Nyamwezi-Kikoba
46	Cantharelloid Clade	Cantharellaceae	*Cantharellus congolensis Beeli*	Hehe-Wisogoro, Nyamwezi-Wingingili, Butoba, Bukukwe mweusi, Turu-Madali, Sumbwa-Mkukwe
47	"	"	*Cantharellus cyanoxanthus* R. Heim ex Heinem	Hehe-Wisogolo; Bena-Bunyamalagata, Wifindi, Nyamwezi-Ungukwe manjano
48	"	"	*Cantharellus isabellinus var. isabellinus* Heinem. Heinem.	Makonde-Chipatwe, Ujama, Upatwe; Mwera-Ubuluwa
49	"	"	*Cantharellus isabellinus var parvisporus* Eyssart. and Buyck	Makonde-Chipatwe, Ujama, Upatwe, Mwera-Ubuluwa
50	"	"	*Cantharellus densifolius Heinem*	Zaramo-Kizogoro
51	"	"	*Cantharellus tomentosus* Eyssart. and Buyck	Makonde-Upatwe mdogo
52	"	"	*Cantharellus rufopunctatus* Heim	Hehe-Wisogoro
53	"	"	*Cantharellus pseudocibarius* Henn.	Hehe-Wisogoro
54	"	"	*Cantharellus subincarnatus* Eyssart. and Buyck	Hehe-Wisogoro
55	"	"	*Cantharellus ruber* Heinem	Kiswahili-Makombo, Lunda-Kadun. Hehe-Wisogoro
56	"	"	*Cantharellus floridulus* Heinem.	Bena-Wigulu, Unyamalagata, Nyambo-Otunyantuku, Nyamwezi-Ungukwe, Hehe-Wisogoro
57	"	"	*Cantharellus rhodophyllus* Heinem	Hehe-wisogoro
58	"		*Cantharellus pseudocibarius* Henn.	Hehe-wisogoro
59	"		*Cantharellus luteopunctatus* (Beeli) Heinem.	Hehe-wisogoro
60	"	"	*Afrocantharellus symoensii* (Heinem) Tibuhwa	Nyamwezi-Mkukwe, Bena-Wifindi, Wisogoro
61	"	"	*Afrocantharellus platyphyllus*	
62	"	"	*Afrocnatharellus splendens* (Buyck) Tibuhwa	Nyambo-Binyantuku
63	"	"	*Afrocantahrellus platyphyllus f. platyphyllus (*Heinem.) Tibuhwa	Bena-Bunyamalagata, Wifindi, Wisogoro
64	"	"	*Afrocnatharellus fistulosus* (Tibuhwa and Buyck) Tibuhwa	Zaramo-kizogoro mdogo, Hehe-wisogoro mdogo
65	Bolete Clade	Boletaceae	*Boletus pallidissimus* Watling	swahili-uyoga msiponji mweupe
66	"	"	*Boletus spectabilissimus* Waltling	swahili uyoga msiponji mwekundu
67	"	"	*Afroboletus luteolus* (Heinem.) Pegler and T.W.K. Young	Swahili-uyogamsiponji mweusi, Bena-windima
68	"	Suillaceae	*Suillus granulatus* (Linnaeus) Roussel	Swahili-usuilis, ngoni-ngowani vitindi
69	Jelly Fungi	Auriculariaceae	*Auricularia delicata (Mont.) Henn.*	Swahili-maghwede laini, Sambaa-maghwede
70	"	"	*Auricularia polytricha* (Mont.) Sacc.	Swahili-manghwede vijisinga, Sambaa-maghwede
71	"	"	*Auricularia cornea* Ehrenb	Sambaa-maghwede
72	"	Tremellaceae	*Tremella fuciformis* Berk	
73	Polyporoid Clade	Polyporaceae	*Polyporus moluccensis* (Mont.) Ryvarden,	Sambaa-ngaha
74	"	"	*Polyporus tenuiculus (P. Beauvois) Fries*	Sambaa-ngaha
75	"	Schizophyllaceae	*Schizophyllum commune* Fr.	Swahili-kipepo uchanga

Among the vital things a mushroom collector must be acquainted with includes: expertise in folk taxonomy, greater knowledge on the habitat niche and morphology of the fungi. Knowing these attributes is very important in determining where to go looking for a certain type of mushroom, of interest and how to handle them in order to lessen their deterioration.

The folk taxa observed in this study were polytypic with the same term used to refer to more than one species (Table 3). This observation concurs with that of Berlin [26,27] who named it as ‘under- differentiation’ and corresponded it with the western scientific classification. This was more evident in the species of the genera *Cantharellus* and *Afrocantharellus*. For example the folk name Wisogoro basically refer to any *Cantharellus*/*Afrocantharellus* growing in miombo woodland by Hehe and Bena tribe except *Cantharellus floridulus* which the Bena refer it as Wigulu/Unyamalagata (Table 3).

Many folk taxa were conceptually distinguished on the basis of very few morphological characters such as color as it has been also observed in Tibuhwa [5]. For example, *Boletus* mushrooms in Swahili language were all referred to as ‘Uyoga msiponji’ meaning sponge mushroom. However, the species were demarcated based on color such as ‘Uyoga msiponji mweupe’, ‘Uyoga msiponji mwekundu’ and ‘Uyoga msiponji mweusi’ for *Boletus pallidissimus*, *Boletus spectabilissimus* and *Boletus spectabilissimus* respectively, which are all white, red and black in color. Swahili is a National language spoken by many tribes in the country. Some of the Swahili folk taxa names were borrowed from specific tribes. A good example is the species in the genus *Auricularia*. In Sambaa *Auricularia* are known as ‘Maghwede’ meaning ‘ears’ as the English name also referred them as ear mushroom due to their shape resembling a normal human ear. The Swahili folk taxa given to these species are Maghwede vijisinga for *Auricularia polytricha* that means ‘ear with spines’ and Maghwede laini for *Auricularia delicata* meaning ear with soft skin with the word ‘Maghwede’ in both names borrowed from Sambaa tribe. This helps in popularizing the folk names as it was noted that different tribes also simply refer them as ‘Maghwede’ (Table 3).

**Figure 3:**
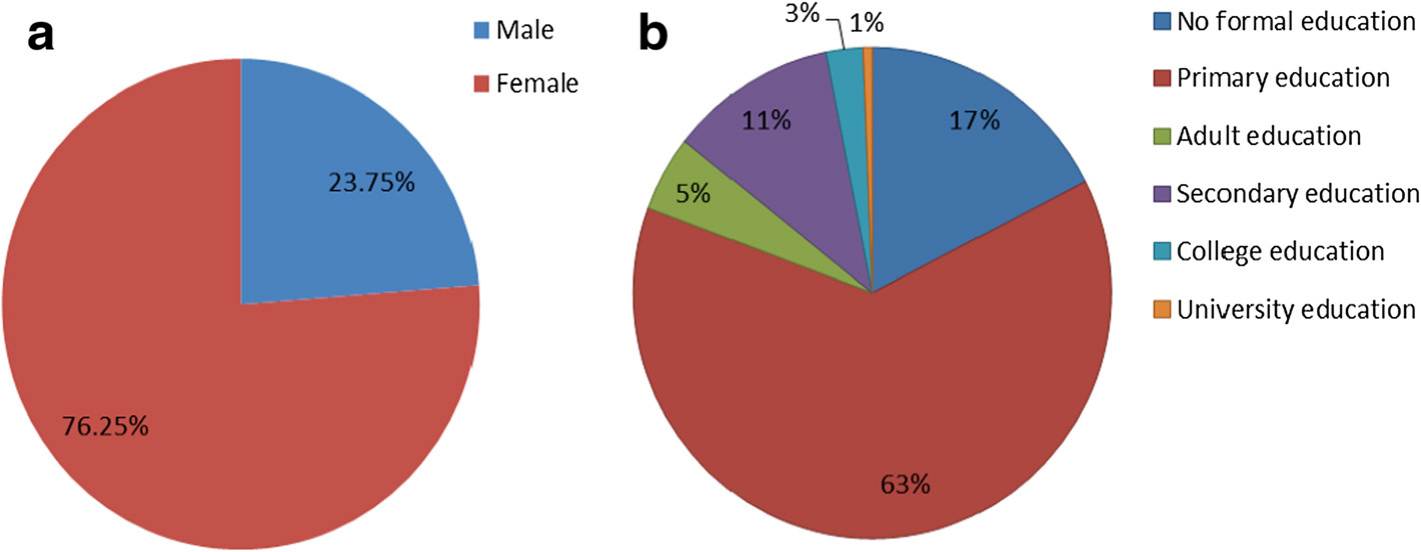
Demographic characters of the studied group showing: (a) Gender participation with women dominating, (b) Level of formal education.

**Figure 4:**
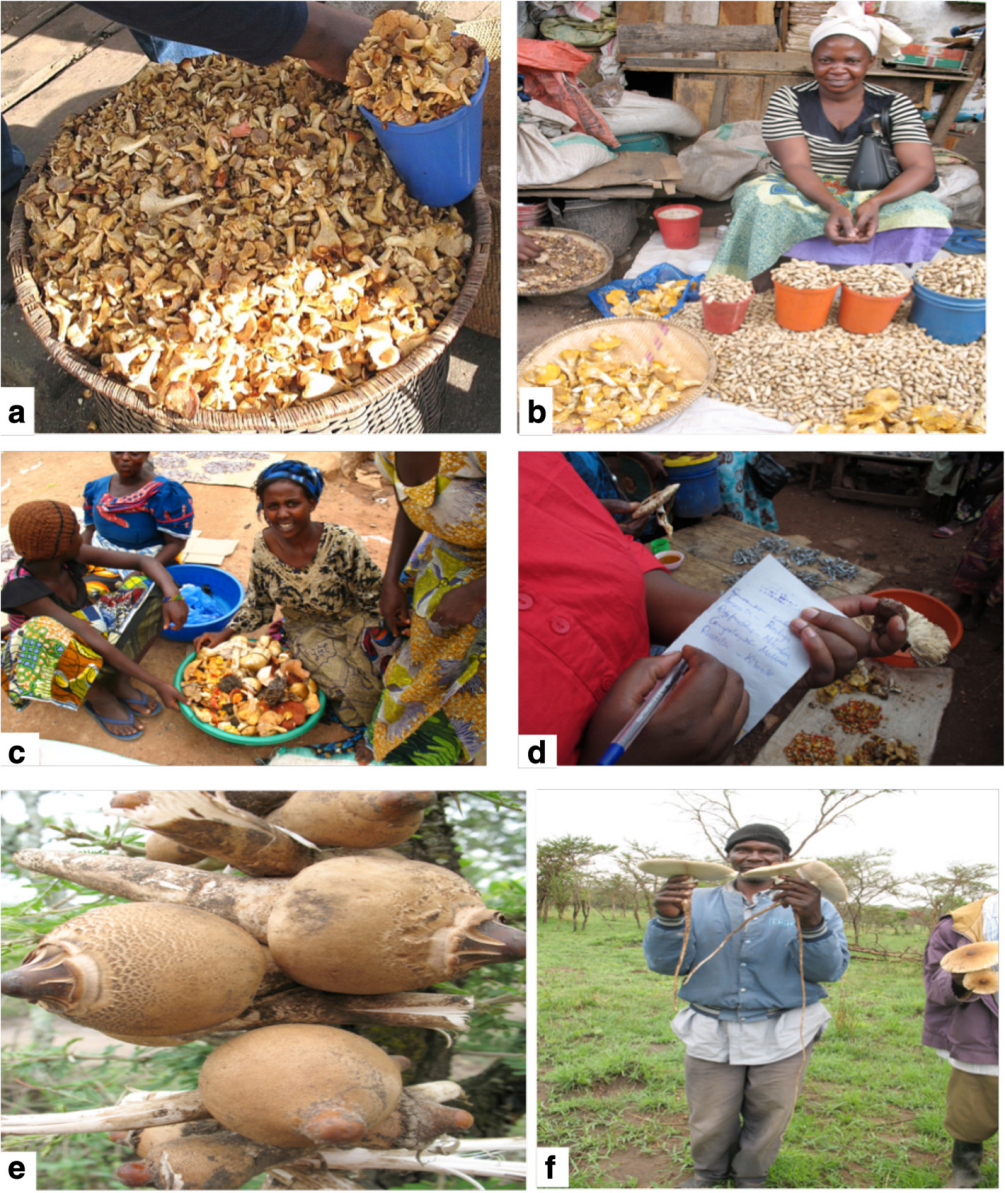
**Field observations of wild edible mushroom presenting: (a) Package for retail selling, Iringa market (b) In Tabora, fresh, dry mushrooms and other cereals (c) In Kigoma a girl selling mixed species of *Cantharellus, Afrocatharellus, Amanita, Russula *** **and *Lactarius *** **(d) The author recording different folk taxa from an interviewee in Kigoma open market (e) Termitomyces le-testui pieced on the string heading to the market in Mara, (f) A man holding *Termitomyces le-testui *** **for the meal in Serengeti.**

Wild mushroom collection seems to be a professional job, requiring long traditional training and acquiring knowledge through experience regardless of the formal education level. The finding reveal that wild mushroom market chain is gender oriented dominated by women (76.25%) while men involvement were only 23.75% (Figure 3). This finding concurs with the observation made by other researchers who also observed that women are the principal mushroom collectors in many parts of the world, playing a central role on mushroom processing both for self-consumption and sale [6,28]. The observed dominance of women in wild edible mushroom market chain in this study implies that, women surely have vast knowledge on mushroom folk taxonomy, edibility, biology, as well as ecological niches. They were thus the principal knowledge transmitter. A recent study by Tibuhwa [5] established that there is a tremendous decline of mycological knowledge with decreasing in age, implying that the traditional mycological knowledge, which basically is the main tool of taxonomy in rural areas, is in danger. There is thus a need to proper document this knowledge, and promote these groups with mycological knowledge by enhancing them to transmitting it to the new generation. Interestingly, when interviewed mushroom collectors who were mainly women were very proud of their knowledge and they value it for its contribution in their subsistence income generation. They were found selling mushrooms in local markets together with other vegetable products such as spinach, carrots, paprika, groundnuts, tomato, and cassava leaves. In Kigoma where they live near Lake Tanganyika also they were selling small fish commonly known as ‘*Dagaa*’ (Figure 4d). To them mushrooms is a source of income and nourishment and contribute largely to their food security and improved livelihood.

During data collection it was also noted that in some places mushroom collectors gain social recognition. In Geita region, when researchers asked about mushroom they were simply directed to ‘*mama uyoga*’ literally means a mother of mushroom. The same was noted in Tabora local market where they simply directed us to ‘*bibi wa uyoga*’ meaning grandmothers of mushroom. Interrogating them they said they become interested in wild mushroom from their childhood when they used to go to the forest areas to collect mushrooms with their parents. However, seven of them confessed that they were taught by their friends as the alternative way of increasing their income and getting delicious food. The social recognition as a mushroom collector has been also observed in other parts of the world. For example, in Central Mexico they are called “*hongueros*” literally means mushroomer [6] while in Hungary they are referred to as “*King of mushrooms*” meaning people with wide knowledge on mushrooms [4].

The local mycological knowledge distribution was found variable within interviewed groups. Those who engage actively in collection were very well knowledgeable than traders and consumers. For example, the collectors were noted to possess more profound knowledge on the biology, ecology, phenology and folk taxonomy of taxa they collected. Noticeably, they were able to explain very certainly as close as to species level different in vernacular languages or the Swahili which is the national language (Figure 4d). The possession of vast knowledge on wild mushroom by collectors has been also documented by Guissou [3] and Zsigmond [4]. Unlike the collectors, traders were not well knowledgeable on the folk taxa they were trading, while consumers were even less concerned on which taxa they consumed. This shows that wild mushroom business can be managed by both people who have lower level of education but with traditional knowledge on folk taxonomy in rural area and those who have no experience in folk taxonomy especially in the trader category. Demographically the old aged group above ≥50 yrs and the low aged group 8–17 yrs were involved in most of wild mushroom collection. While the trader category comprised mixture of all age group but dominated by middle-aged group (Table 2). Based on these results, it is clear that wild mushroom resources can provide employment opportunities for all age groups including old people aged above 50 yrs.

### Why harvesting wild mushrooms? Testimonies from collectors

In a nutshell, the only capital investment involved in wild mushroom harvesting is the energy spent to gather the mushroom and small buckets and sacs used in carrying harvested mushroom back home. This fact makes this economic activity of its own kind. Many disadvantaged group in rural areas especially old people, women and children, they see it as a source of quick money. Below are five selected testimonies from different wild mushroom collectors.

● One old lady claimed that ‘*she likes picking mushroom because they earn her easy money. She proudly say that the activity do not cost her a lot of time and energy compared to cultivating cereals and vegetables which involve tilling the land, sawing, weeding, waiting for them for several months to ripen’.*

To her, wild mushroom are just ready made products for food and selling.

● One middle aged lady said “*Rain season is money generation time’ I only have to be well, it takes me only two hours to pick about one bucket of mushroom measuring 20 liters from miombo wood land which earn me about 10–15 US dollars depending on the market demand of the day”.*

To her rain season is harvesting time and economic growth period.

● One of the middle aged collector testified that *due to excessive drought agriculture has become less profitable, thus forcing them to find other means for earning a living, for example, collecting and selling wild mushrooms.*

To her, collect wild mushroom is an immediate alternative solution for earning income after being disappointed by prolonged drought, which affected her cultivated crops.

● One children aged 12 said that *accompanying his grandmother in mushroom gathering is good not only the activity provide him with him a delicious meal but also when they sell some, his grand mother give him some money to buy his school needs like text book and pen.*

To him, wild edible mushroom gathering provides him with school needs and delicious meals.

From these testimonies, it implies that mushrooms are often an economic alternative for most disadvantaged groups, such as widows with young children or women who are the head of their families. This impression has been also observed in local populations of the rainforest of south Cameroon by Van Dijk *et al*. [29] and established in ethnomycological review study by Garibay-Orijel *et al*. [6].

Responding to a question, How many years are you involved in the wild mushroom business? The wild collectors, who were the majority of the whole sellers (65%), expressed that they had an experience of 5 years and above. It was, however, revealed that majority of the retail sellers (72%) had less experience of 1–3 years showing that probably retail sellers do not involve in the open market business for a long time. They probably do that in order to increase their capital before they quit to other higher business.

### Methods employed in mushroom hunting/Experience in wild mushroom collection

In mushroom gathering, locating a particular species is more of a challenge facing the collectors. However, they usually forage in fixed “paths” or forest areas. These strategies enabled them to maximize the chance of finding a group of species at a given time of the year. For example, communities living near miombo woodland, they know exactly where to harvest highly priced *Cantharellus*, *Afrocantharellus* and some *Amanita* species. On the other hand, those living in tropical indigenous forest know specific trees and termite mounds as well as the season of the year specific mushroom taxa fruit out. The wild mushroom collection techniques were similar involving visiting different forest sites during the rain season for collecting mushrooms in their buckets and sacs except for the large mushroom of the *Termitomyces* genera. In this genus, different communities testified that, gatherers walk around during early rains and wherever they see signs of termite on or near the termite mounds, they put special signs. The signs symbolize to the rest of the community that they already booked for the coming mushroom and culturally the rest members of the community do respect a booking sign. Interestingly, it was observed in this study that the mostly harvested species are mycorrhizas belonging to genera, *Cantharellus*, *Lactarius, Termitomyces, Russula* and *Amanita* (Table 3) not currently cultivated and their fruit bodies are scarce. Few saprophytic mushrooms including members of the genus *Pleurotus, Aulicularia, Armillaria, Coprinus* and *Agaricus* were also noted (Table 3). Some of these saprophytic species such as *Pleurotus* and *Coprinus* the technical knowledge for their cultivation is available in the country, thus can be artificially cultivated [30-32].

### Processing and preservation for short and long-time utilization

In this study different methods were found used by different communities for both short and long term preservation to ensure all year supply of mushrooms. In the local open markets, both fresh and dry mushroom were sold (Figure 4d). The dry mushrooms were sold at relatively higher prices compared to fresh mushrooms. Interrogating the collectors and traders, different ways deployed in improving the shelf life of the collected mushrooms were revealed as follows:

#### *Fresh preservation*

This involved soaking them in water where they remained fresh for 2–3 days. These methods were observed in areas with relative cool temperature of 15-18°C in Lushoto- Tanga. Thinking about this method, the mushroom remain fresh simply because waters in these cooler areas are really cold, thus soaking mushroom in these water reduces the biological activity of the mushrooms as the same principle used in storing them in the fridges. In warmer areas like in Dar es Salaam, Tabora and Kigoma, they just spread the mushroom on the ground or mattress from collecting buckets and sacs and leave them outside the house over night before transporting them to the market.

#### Long preservation

***Sun drying ***This involved direct sun drying whereby mushroom were spread on the ground/wire meshed shelves and left them to dry by direct sunshine. This method is the best and has been recently found to enhance increased antioxidant activities in *Coprinus* mushrooms [32]. Sun drying and keeping them in airtight container/plastic bags can stay for over one year in good condition.

***Smoking ***This involves preserving the collected mushroom on shelves constructed above the cooking premises. In rural area, cooking is done using firewood which gives out heat and smoke that goes straight to the mushroom preserved on the shelves constructed above the cooking points. The heat help in drying the mushroom, while smoke impart some chemicals to the mushrooms including formaldehyde which has preserving effect although it has recently been associated with carcinogenic properties [33]. Smoked mushroom can stay up to three years in good conditions and consumer testified those smoked mushrooms are very delicious and tasty.

#### *Salt drenching*

This involves preserving mushroom in a supersaturated sodium chloride solution. This goes without a question that the saline condition kills most of the microbes that would have caused mushroom deteriorations thus remain in good condition. The method was mostly observed in Kigoma - Uvinza where they have salt panels in the area. Discussing the applicability of this method with other interviewee in other parts, they were doubtful on the method as it could be expensive since it will involve spending money for buying salt.

### Wild mushroom and healthy food

Tanzania is among developing countries thus most of her people especially those living in rural areas cannot get adequate intake of essential food with balanced food rich in essential compounds such as proteins, vitamins, minerals and essential fatty acids. Wild edible mushrooms have these essential compounds and functional substances for human health including bioactive components including phenols, flavonoid, β-carotene, lycopene β-glucans and Vitamins [32,34,35]. It is well document in FAO reports that undernourishment is a characteristic feature of poverty and a direct violation of a universally recognized human right. Undernourishment poses lots of negative effects to human which lead to illness in people including underweight new born babies thus face nutritional handicap that affect their health structure throughout their lives [36]. Collecting and eating wild mushroom in rural areas not only will provide them with a delicious food, but also a healthy one.

### Wild mushroom collection and forest ecosystem conservation

Some poor families in villages are even more disadvantaged by having no enough land to produce crops and raise animals. Nevertheless, the available fertile land in some places is kept under rural environment protection agency, for the purposes of protecting forest ecosystems which in turn denies the right of the poor famers to assess it. This has been causing a lot of conflicts between rural dwellers and the authorities [37-41]. Among the problem that faces whole seller traders who are mainly wild mushroom collectors is some restriction to get into conserved forests. Mushrooms are fruit bodies of certain groups of fungi, thus collect mushroom fruit bodies will under normal circumstances pose little effect to the fungus itself, since only the fruits are being harvested. This study thus calls for good cooperation and general awareness to forest conservers to freely allow mushroom gatherers to collect and use these underutilized resources from the forest, which hardly affects the conserved forest. It is also clear that if communities will be allowed to harvest these mushroom resources they will in turn be cooperative in preserving these forests since they will have tangible direct benefits from them thus reduces community conflicts with environmentalist and forest ecosystem conservers. Moreover, the study promotes a purposeful awareness to rural dweller, to harvest and utilize this underutilized local resources, which not only will provide with them nutritious food but also an alternative employment opportunities in rural areas especially to the disadvantaged groups women and old people.

### Economic aspects of collecting wild edible mushrooms

The observed average harvests for each season collections were 20–30 buckets per season. The observed market price ranged from $10–15 per bucket, which would earn a picker about $200–450 per season. Since Tanzania experience two rain seasons (the long rains of March- May and short rains of October- December), one can thus annually earn about $400–900. This data was mainly true to the societies which live near the miombo woodland especially in Tabora, Iringa, Geita and Kigoma (Figure 1). Miombo woodlands are dominated by the mycorrhiza trees which live symbiotically with fungi of the group Basidiomycetes and are ectomycorrhiza thus fruit out during the rainy season [24]. The dominant genera of edible species collected from these woodlands belongs to *Cantharellus, Afrocantharellus, Lactarius, Rusulla, Amanita* and *Boletus* (Table 2) which are mainly sold in mixture along the road sides and local markets (Figure 4). The results also consistently revealed the overrepresentation of women, unemployed; people who are comparatively older, less educated and retired on participation in wild mushroom market chain (Figure 3b, Table 2). This finding is partly in line with that of Cai *et al*. [16] who observed more representation of women, retired and less- educated but not with unemployed people in Eastern Finland wild edible fungi industry. Society development necessities fostering pro-poor economical growth and enhance poor people to access important services which help in eradicating poverty [35]. In order to eradicate undernourishment and build a healthy community with high working capacity income growth of the society is essential. Wild mushroom collection not only provide nutritious food, but also can in turn be sold and contribute to family income. For example, it is well document that WEM harvesting in developed country is among the multimillion dollar industries [39]. In this study it was revealed that a wild mushroom collector can generate up to $400–900 thus allow them to live an improved life compare to the gross national income (GNI) per capita estimated at $340 annually [17]. Tanzania being rich in tropical forests and miombo woodland where the WEM grow abundantly [24,42] it is very important the responsible authority upgrade this underutilized resource for the betterment of the society and nation at large.

## Conclusion

The discussion in this study represents a significant contribution to the field of ethnomycology and wild mushroom contribution to the marginal rural economy in the country. Wild mushroom harvesting not only provides to the rural dwellers with healthy food, but also it brings economic benefits to unemployed people in these sidelined areas. It also revealed that utilizing this resource posse little effect to conserved land forest, thus allowing the nearby community to exploit it might reduce community conflicts with conservers, as they will have the direct benefits from conserved forests. It is therefore anticipated that, this study will serve to stimulate more study in this fascinating area of research while awakening the responsible authorities to promote utilization of these underexploited valued resources.

## Abbreviations

WEM: Wild edible mushroom.

## Competing interests

The author declares that she has no competing interests.

## Authors’ information

This manuscript has been prepared by a single author following 14 years of active engagement in mushroom research in Tanzania.
